# Coping with stress during the COVID-19 pandemic in the oldest-old population

**DOI:** 10.1007/s10433-022-00719-w

**Published:** 2022-08-11

**Authors:** Sina Kathrin Gerhards, Alexander Pabst, Susanne Röhr, Melanie Luppa, Steffi G. Riedel-Heller

**Affiliations:** 1 Medical Faculty, Institute of Social Medicine, Occupational Health and Public Health (ISAP), University of Leipzig, Philipp-Rosenthal-Straße 55, 04103 Leipzig, Germany; 2 Global Brain Health Institute (GBHI), Trinity College Dublin, Dublin, Ireland

**Keywords:** Mental health, Public health, Old age, COVID-19 pandemic, Coping, Stress

## Abstract

The aim of the study is to investigate psychosocial factors that are associated with positive and negative coping with stress, as well as with worries about and perceived threat by COVID-19 to enable us to provide adequate support for oldest-old individuals. A paper–pencil-based survey assessed COVID-19 worries and perceived threat, depression, anxiety, somatization, social support, loneliness, resilience, positive and negative coping in a sample of *n* = 197 oldest-old individuals (78–100 years). Linear multivariate and binary logistic regression analyses were conducted. Individuals with high levels of resilience were more likely to feel self-efficient when coping with stress. High levels of depression, anxiety and loneliness were associated with feeling more helpless when coping with stress. However, oldest-old individuals who felt lonely also experienced situations where they felt competent in stress coping. Being male and experiencing high levels of social support was more likely associated with high levels of worries due to COVID-19. Increased age and higher levels of depression were associated with lower levels of perceived personal threat, whereas higher somatization scores were more likely associated with higher perceived personal threat. Findings suggest that mental health factors may shape the way oldest-old individuals cope with pandemic-related stress. Resilience might be an important factor to take into account when targeting an improvement in positive coping with stress. Oldest-old individuals who have higher levels of depression, anxiety and feel lonely may be supported by adapting their coping skill repertoire to reduce the feeling of helplessness when coping with stress.

## Introduction

In Germany, the first case of the coronavirus disease COVID-19 caused by the SARS-Cov-2 was reported in January 2020. The outbreak of the disease was closely followed by strict mass quarantine measures by the government trying to curb the spread of the disease. The elderly population was identified as high risk group for a severe course of disease and high mortality (Robert Koch Institute 2020; Verity et al. 2020). Multiple sources of problems arose especially for the elderly, like fear of infection with the virus, social distancing and isolation, worries about the health of loved ones and worries about the uncertain course of the pandemic, potentially causing high psychological distress (Taylor et al. 2020).

In the beginning of the pandemic, first studies suggested that the older people’s mental health remained roughly stable with some people reporting having high levels of perceived threat (Röhr et al. 2020). What we do not know yet, and this is important since experiencing distress because of the pandemic is associated with a higher risk for developing mental health problems (Schnell & Krampe 2020), is how the oldest-old population experiences the pandemic and how they feel when coping with pandemic-related stress.

We know from previous studies with adults that the way one copes with adverse and challenging events like the COVID-19 pandemic is important for one’s general mental well-being (Lopez et al. 2020; Tomás et al. 2012). There is also research showing that anxiety and depression are linked with a more dysfunctional way of dealing with COVID-related stress in an adult sample of 18–76-year-old people (Kar et al. 2021). Furthermore, higher levels of mental distress, e.g. depression and anxiety, are associated with more negative ways of coping with stress (Li et al. 2012; Orgeta & Orrell 2014; Raut et al. 2014), which in turn is associated with poorer cognitive functioning (Aggarwal et al. 2014; Wilson et al. 2007) and higher risk for dementia (Wilson et al. 2007). Positive coping on the other hand is associated with lower levels of anxiety, hopelessness and loneliness (Oles et al. 2014), leading to better brain health (Arenaza-Urquijo et al., 2020). Those findings stress the importance to investigate how the oldest-old population deals with the pandemic-related stress, but to our knowledge, it has not yet been explored in the German oldest-old population.

A factor that may be associated with a positive way to cope with COVID-19-related stress is the extent of perceived resilience. Studies have shown that resilience is positively related to adaptive, active coping strategies that in turn lead to less perceived distress and lower cortisol levels (Smith et al. 2016; Vannini et al. 2021). With increased age resilience might increase since the older individual has more life experience in mastering challenging events (Hardy et al. 2004; Welzel et al. 2021).

In the current study, we aim to close this existing gap in literature by examining the association of sociodemographic variables, mental well-being, loneliness, social support and resilience with positive and negative coping with stress and with COVID-19-specific worries and perceived personal threat by the virus, with the overall aim to identify mental and social health factors that make an individual of old-age specifically vulnerable. This knowledge will not only help during the ongoing pandemic but also enable us to support the very old population in coping with general adverse life events that they have to face with increased age, for example the loss of close ones. Deduced from the before mentioned literature, we assume

- that oldest-old participants with higher depression and anxiety scores show higher ratings on the negative coping scale that is feeling more helpless when coping with stress and lower ratings on the positive, self-efficient coping scale, and
- that higher perceived resilience is associated with less negative, helpless coping with stress and more positive, self-efficient coping with stress due to the pandemic in the oldest-old population.

The associations of sociodemographic characteristics and social health factors with perceived coping ability (positive and negative), as well as the associations with experienced distress (worries and perceived personal threat) due to the pandemic are investigated exploratively.

## Methods

### Setting, study design and sample

From 8 May to 7 June 2020, 378 community dwelling late elderly people aged 78 and older were contacted and asked to take part in this observational study that was conducted with a paper–pencil-based assessment. The assessment was done during the first wave of the COVID-19 pandemic that lasted from March to July 2020 and participants live in the city of Leipzig, Germany (over 600.000 inhabitants in 2021), and its close environs. Governmental measures to curb the virus by closing restaurants, schools and universities and national curfews and restriction of contacts became effective starting March 2020 (see e.g. Khairulbahri 2021). The sample was drawn from the institute’s databank of potential participants who took part in previous population- and primary care-based old age studies and agreed to being contacted for future study purposes. The study has been approved of by the Ethics Committee of the Medical Faculty of the University of Leipzig (ethic approval number: 206–20-ek). All participants were informed about the study’s purpose and gave their written consent. They were not compensated for participation.

### Measures

#### Independent variables

Collected sociodemographic data included age (years), gender (male/female), education (low/middle/high), marital status (married/ single or divorced/ widowed) and living situation (alone/ with partner or others).

The Brief Symptom Inventory (BSI-18; Derogatis 1993) was used to assess the symptomatology of depression, anxiety and somatization during the past seven days using the same-named subscales consisting of six items each. The items can be answered using a five-point Likert scale (“not at all” to “very strongly”, scored 1 to 5). The Brief Symptom Inventory has proven to have good psychometric properties (Franke et al. 2017). In our study, the subscales show good reliability with Cronbach’s alphas of *α* = 0.70 for the somatization scale, *α* = 0.73 for the anxiety scale and *α* = 0.77 for the depression scale.

The German Version of the ENRICHD Social Support Inventory (ESSI-D; Kendel et al. 2011) was used to assess the subjective experience of social support and consists of five items that can be rated on a five-point Likert scale (“never”, “seldom”, “sometimes”, “often”, “always”; scored 1 to 5). Mean scores were calculated with higher scores indicating higher social support. In the current study, the scale shows very good reliability with a Cronbach’s alpha of *α* = 0.90.

To measure loneliness, the three-item version of the University of California, Los Angeles Loneliness Scale (UCLA-3) was used (Russell et al., 1980). Three items can be rated on a four-point Likert scale (“often”, “sometimes”, “seldom”, “never”, scored 0 to 3). Mean scores were calculated for further analysis. The scale has shown good internal consistency as well as discriminant and concurrent validity (Hughes et al., 2004). The scale shows good reliability (*α* = 0.76) in our study as well.

Resilience was measured by the validated German version of the Brief Resilience Scale (BRS; Chmitorz et al. 2018). Six items that can be rated on a five-point Likert scale from “totally disagree to “totally agree” assessed the person’s ability to rally from stress, respective resilience. To reduce response bias in terms of social desirability, three items of the questionnaire are negatively and three items positively worded. To quantify resilience, the mean score of all items was calculated with higher scores indicating higher resilience. Principal component analyses supported the bi-factorial structure of the questionnaire as previously described by Chmitorz et al. (2018). In line with the author’s suggestion, we estimated and adjusted for a method factor. The Cronbach’s alpha in our study is *α* = 0.84.

#### Dependent variables

Participant’s perceived stress and the coping with it during the past month was measured by the Perceived Stress Scale 4 (PSS-4; Cohen et al. 1983) that consists of four items that can be rated on a five-point Likert scale (“never” to “very often”, scored 1 to 5). Principal component analysis confirmed the two-dimensional factor structure of the questionnaire that was described in previous studies (Leung et al. 2010). One factors includes the item “In the last month, how often have you felt that you were unable to control the important things in your life?” and “In the last month, how often have you felt difficulties were piling up so high that you could not overcome them?”. The other factor includes the item “In the last month, how often have you felt confident about your ability to handle your personal problems?” and “In the last month, how often have you felt that things were going your way?”. The first factor is measuring the ability to cope with stress in a positive, self-effective way (here: “positive coping”), and the second factor is assessing the negative affective component of coping with stress resulting in helplessness (here: “negative coping”; the description of the factors is in line with previous research, e.g. Bastianon et al. 2020). The Perceived Stress Scale has shown good reliability (Kunzler et al. 2018; Leung et al. 2010; Warttig et al. 2013). The scale shows acceptable reliability in our study with a Cronbach’s alpha of *α* = 0.65 for the positive subscale and *α* = 0.66 for the negative subscale.

Participants were asked single item questions that were directly related to the COVID-19 pandemic and specifically created for the purpose of the study assessing general worries about the COVID-19 pandemic and the perceived threat by COVID-19. Items were phrased as statements (“I am worried about the coronavirus”, “I feel personally threatened by the coronavirus”) and were rated on a five-point Likert scale (“strongly agree” to “strongly disagree”, scored 0 to 4). Scores were classified as low (score between 0 and 2) or high (score of 3 or 4).

### Data analysis

Statistical analyses were conducted using STATA 16.0 SE (College Station, Texas, USA). First, descriptive statistics for all sociodemographic, psychosocial and COVID-related parameters were calculated. To identify gender differences, Chi-square (*x*^2^) tests and Wilcoxon rank-sum (Mann–Whitney) tests were calculated for all sociodemographic and psychosocial variables, as well as variables concerning worries about and perceived personal threat by COVID-19. In a next step, two linear multivariate regression models were conducted to predict positive coping with stress and negative coping with stress by including sociodemographic factors, somatization, depression, anxiety, social support, loneliness and resilience in the models. The scale’s residual distributions were inspected with skewness and kurtosis values within the acceptable ranges (skewness between − 2 and + 2; kurtosis between -7 and—+ 7, see e.g. Cohen et al. 2002). Furthermore, two binary logistic regression models were fitted to assess associations of sociodemographic characteristics, somatization, depression, anxiety, social support, loneliness and resilience with worries about COVID-19 and perceived threat by COVID-19. The variables included in the models were continuous, except for gender (male in reference to female), marital status (single/divorced, widowed in reference to married), living situation (living alone in reference to living with partner or other), education (categorized according to Comparative Analysis of Social Mobility in Industrial Nations/CASMIN classification as middle, high in reference to low). For the linear multivariate regression models predicting positive and negative coping with stress, we additionally reported standardized beta values ($\beta )$ to allow for direct comparison between the variables. We used clustered standard errors to account for heteroscedasticity across the observations since participants originally came from different studies. Most important sample size criteria for conducting the regression analyses were met. With an assumed *R*^2^ of 0.15, a statistical power of 0.8 and a significance level of *α* = 0.05, one would need a sample size of *n* = 110 for our models. With an assumed *R*^2^ of 0.10, one would need a sample size of *n* = 162. With *n* = 182 for the coping with stress model and *n* = 177 for the model predicting worries and perceived threat due to COVID-19, we fulfilled this criterion.

## Results

### Sample characteristics

Out of *n* = 378 contacted people, *n* = 197 (52.12%) agreed to take part in the study and send back their filled out questionnaire. The participants’ age range was 78 to 100 years (mean = 87.88, SD = 4.88) with 40.1% being male and 59.9% being female. More sociodemographic characteristics of the sample can be retrieved from Table 1 that shows the information subdivided by the total sample, women and men. Additionally, significance levels are reported to identify gender differences. Compared to men, women were slightly less educated (high education level: 26.6% vs. 48.7%; *x*^2^ = 9.754, *p* = 0.008), less often married (29.1% vs. 66.7%; *x*^2^ = 26.91, *p* < 0.001) and more often living alone (62.1% vs. 28.6%; *x*^2^ = 20.79, *p* < 0.001).

**Table 1:** Sociodemographic characteristics of the study sample (*n* = 197)

	Total	Women (*n* = 118)	Men (*n* = 79)	Group difference (*p*-value)^1^
Age; M (SD, range)	87.88 (4.88, 77.68–99.92)	87.71 (5.05)	88.12 (4.64)	.471
*Education; n (%)*				
Low	67 (35.4)	46 (40.7)	21 (27.6)	.008
Middle	55 (29.1)	37 (32.7)	18 (23.7)	
High	67 (35.4)	30 (26.5)	37 (48.7)	
*Marital status; n (%)*				
Married	86 (44.1)	34 (29.1)	52 (66.7)	< .001
Single/divorced	19 (9.7)	14 (12.0)	5 (6.4)	
Widowed	90 (46.2)	69 (59.0)	21 (26.9)	
*Living situation; In (%)*				
Living alone	94 (48.7)	72 (62.1)	22 (28.6)	< .001
Living with partner/others	99 (51.3)	44 (37.9)	55 (71.4)	

Missing values: education: *n* = 8 (4.1%); marital status: *n* = 2 (1.0%); living situation: *n* = 4 (2.0%); ^1^Group differences were calculated using Wilcoxon rank-sum (Mann–Whitney) for age and chi-square tests for education, marital status and living situation

Descriptive statistics of all psychosocial variables used in the current study are shown in Table 2 with mean scores (*M*) and standard deviations (*SD*), again presented subdivided by the total sample, women and men. Women reported slightly higher levels of negative coping with stress, thus felt more helpless, compared to men (*M* = 2.46, SD = 0.98 vs. *M* = 2.10, SD = 0.79; *z* = 2.363, *p* = 0.018). Women were also slightly more depressive compared to men (*M* = 1.41, SD = 0.47 vs. *M* = 1.28, SD = 0.38; *z* = 2.041, *p* = 0.041).

**Table 2:** Mental and social conditions during the COVID-19 pandemic of the oldest-old population (*n* = 197); mean (SD)

	Total	Women (*n* = 118)	Men (*n* = 79)	Group difference (*p*-value)^1^
Social support (ESSI)^a^	4.36 (0.85)	4.28 (0.91)	4.48 (0.85)	.277
*Psychological stress (PSS-4)*^*b*^				
Positive coping	3.20 (0.95)	3.14 (0.92)	3.29 (0.98)	.162
Negative coping	2.32 (0.92)	2.46 (0.98)	2.10 (0.79)	.018
*Psychological burden (BSI-18)*				
Somatization	1.73 (0.57)	1.76 (0.56)	1.68 (0.58)	.196
Depression	1.36 (0.44)	1.41 (0.47)	1.28 (0.38)	.041
Anxiety	1.35 (0.37)	1.38 (0.39)	1.31 (0.33)	.454
Loneliness (UCLA)^d^	1.14 (0.81)	1.22 (0.84)	1.02 (0.76)	.102
Resilience (BRS)^e^	3.04 (.67)	3.34 (0.67)	3.51 (0.67)	.096
*Worries about the Corona Virus*^*f*^*; n (%)*				
Low	85 (45.2)	55 (49.1)	30 (39.5)	.193
High	103 (54.8)	57 (50.9)	46 (60.5)	
*Perceived threat by COVID-19*^* g*^*; n (%)*				
Low	110 (58.5)	69 (61.6)	41 (53.9)	.296
High	78 (41.5)	43 (38.4)	35 (46.1)	

^a^ESSI = ENRICHD Social Support Scale, ^b^PSS-4 = Perceived Stress Scale: positive and negative subscale, ^c^BSI-18 = Brief Symptom Inventory: somatization, depression and anxiety subscales, ^d^ UCLA = University of California Los Angeles Loneliness Scale, ^e^ BRS = Brief Resilience Scale. Missing values: ^f,g^*n* = 9 (4.6%)

^1^ Group differences were calculated using the Wilcoxon rank-sum (Mann–Whitney) and chi-square tests as appropriate

### COVID-19-specific measures

Furthermore, Table 2 shows the response behaviour for the COVID-19-specific questions. Participants reported their worries about COVID 19 with about half of them (45.2%) showing low levels of worries and the other half (54.8%) showing high levels of worries regarding COVID-19. When being asked about their perceived threat by COVID-19, almost half of the participants (41.5%) reported experiencing high levels of threat by COVID-19. There were no gender differences.

### Factors associated with positive and negative coping with stress

The model of factors associated with positive coping explained 19% of variance (see Table 3). The highest effect can be attributed to resilience (*β* = 0.398, *p* = 0.020) with higher resilience being associated with higher levels of positive coping. There is also a significant effect of loneliness with higher levels of loneliness being associated with slightly more positive coping styles (*β* = 0.155, *p* = 0.026). Participants with a higher education level scored slightly higher on the positive coping with stress scale compared to participants with a lower education level (*β* = 0.188, *p* = 0.005).

**Table 3:** Linear multivariate regression models predicting positive and negative coping with stress (*n* = 182)

	Positive coping with stress			Negative coping with stress		
	b [95%-CI]	$\beta$	*p*	b [95%-CI]	$\beta$	*p*
Gender						
Female (ref.)						
Male	.15 [.00, .29]	.08	.051	− .29 [− .44, − .13]	− .15	.010
Age	.01 [− .07, .09]	.04	.780	.02 [.00, .04]	.10	.077
Marital status						
Married (ref.)						
Single/divorced	− .16 [− .68, .37]	− .05	.410	.31 [− .70, 1.32]	.10	.402
Widowed	− .03 [− .64, .58]	− .02	.884	− .08 [− 1.02, .86]	− .05	.794
Living situation						
Living with someone (ref.)						
Living alone	.29 [.41, .99]	.16	.276	− .10 [− .68, .49]	− .05	.640
Education (CASMIN)						
Low (ref.)						
Medium	.09 [− .50, .68]	.04	.658	− .32 [− .58, − .06]	− .16	.029
High	.37 [.21, .52]	.19	.005	.01 [− .14, .16]	.00	.919
Somatization	.21 [− .34, .77]	.13	.306	.17 [− .13, .47]	.10	.166
Depression	− .33 [− .84, .17]	− .16	.126	.37 [.28, .46]	.18	.001
Anxiety	.18 [− .18,.53]	.07	.217	.57 [.47, .68]	.23	< .001
Social support	.13 [− .05, .30]	.11	.114	.10 [− .07, .28]	.09	.166
Loneliness	.18 [.04, .31]	.16	.026	.27 [.00, .54]	.24	.048
Resilience	.56 [.17, .94]	.40	.020	− .01 [− .48, .45]	− .01	.930
Method factor	− .12 [− .42, .17]	− .11	.277	− .07 [− .32, .18]	− .07	.435
*R*^2^	.191			.424		

Table 3 also shows that the variables included in our model associated with negative coping with stress explained 42% of variance. Men showed less negative coping with stress compared to women (*β* = − 0.153, *p* = 0.010), and a medium level of education was associated with slightly less negative coping compared to low education levels (*β* = − 0.157, *p* = 0.029). Depression and anxiety significantly contributed to explained variance in negative coping with stress with a higher extent of depressive (*β* = 0.175, *p* = 0.001) and anxiety symptoms (*β* = 0.225, *p* < 0.001) being associated with more negative coping. Perceived loneliness is associated with negative coping with stress with higher levels of loneliness being associated with higher levels of negative coping (*β* = 0.242, *p* = 0.048).

### Factors associated with COVID-19-specific worries and perceived personal threat by COVID-19

In Table 4, the results of the binary logistic regression analysis are presented. The model explained 14.8% of variance in perceived worries due to COVID-19. Men tend to show higher levels of worries about COVID-19 compared to women (*OR* = 1.75, *z* = 4.83, *p* < 0.001), and higher levels of social support are more likely associated with high worries about COVID-19 (*OR* = 1.82, *z* = 3.79, *p* < 0.001).

**Table 4:** Binary logistic regression models predicting COVID-19 worries and perceived threat by COVID-19 (*n* = 177)

	Worries about COVID-19				Perceived personal threat by COVID-19			
	OR	95% CI	*z*	*p*	OR	95% CI	*z*	*p*
Gender								
Female (ref.)								
Male	1.75	[1.39, 2.20]	4.83	< .001	1.47	[.84, 2.57]	1.36	.175
Age	.95	[.89, 1.03]	− 1.26	.207	.87	[.80, .95]	− 3.11	.002
Marital status								
Married (ref.)								
Single/divorced	.86	[.06, 11.57]	− .011	.909	.70	[.23, 2.11]	− .064	.524
Widowed	1.32	[.10, 17.87]	.021	.834	1.52	[.29, 7.93]	.50	.619
Living situation								
Living with someone (ref.)								
Living alone	.91	[.14, 5.73]	− .11	.914	.89	[.14, 5.69]	− .13	.898
Education (CASMIN)								
Low (ref.)								
Medium	1.57	[.44, 5.58]	.070	.485	1.00	[.20, 5.05]	.00	.997
High	1.78	[.81, 3.94]	1.43	.153	1.45	[.96, 2.20]	1.78	.075
Somatization	1.24	[.82, 1.86]	1.03	.305	1.38	[1.29, 1.47]	10.02	< .001
Depression	1.00	[.71, 1.42]	.02	.983	.42	[.20, .91]	− 2.20	.028
Anxiety	1.30	[.58, 2.89]	.64	.522	2.21	[.26, 18.53]	.73	.466
Social support	1.82	[1.33, 2.47]	3.79	< .001	1.12	[.67, 1.87]	.45	.656
Loneliness	1.15	[.97, 1.36]	1.59	.113	.77	[.44, 1.36]	− .90	.370
Resilience	.98	[.53, 1.81]	− .07	.943	.87	[.33, 2.32]	− .27	.785
Method factor	.69	[.25, 1.89]	− .73	.468	.70	[.46, 1.07]	− 1.66	.097
Nagelkerke pseudo *r*^*2*^	.148				.174			

The model investigating associations of psychosocial factors with perceived personal threat by COVID-19 explained 17.4% of variance. With increasing age, participants tend to be less likely to experience high perceived threat by COVID-19 (*OR* = 0.87, *z* = -3.11, *p* = 0.002). Somatization was associated with a slightly higher chance of perceiving high threat by COVID-19 *(OR* = 1.38, *z* = 10.02, *p* < 0.001). Finally, a higher extent of depressive symptoms is more likely associated with low perceived threat by COVID-19 (*OR* = 0.42, *z* = − 2.20, *p* = 0.028).

## Discussion

In the current study, we aimed to examine the association of sociodemographic and mental health factors, social support, loneliness and resilience with positive and negative coping with stress in the oldest-old population. We also investigated the associations of the before mentioned variables with COVID-19-specific worries and perceived personal threat by the SARS-COV-2 virus.

### Coping with stress

Resilience had the highest effect on positive coping with stress in our sample of the very old population, and this supports our hypothesis that high perceived resilience is associated with more positive coping. This is also in line with previous findings that suggest that resilience plays an important role in adaptive coping and feeling competent and self-efficient in coping with stress (Tagay et al. 2016; Vannini et al. 2021). This further underlines the importance to include resilience training in interventions targeting self-efficient coping and, consequently, better mental health in the very old population during the pandemic.

When taking a closer look at negative coping resulting in a feeling of helplessness, anxiety and depression may be important factors to look at. Participants with higher levels of anxiety and depression were also more likely to feel helpless in their stress coping more often than participants with lower anxiety and depression levels. This supports our hypothesis that high depression and anxiety levels are associated with more negative, helpless coping. Since feeling hopeless and helpless is one of the features of depression, this feeling seems to reflect on the perceived competence in coping with stress. Our finding is also consistent with the findings of the review by Li et al. (2012) who found that anxiety and depression are linked to more dysfunctional coping strategies. Kar and colleagues (Kar et al. 2021) found that anxiety and depression are linked to a struggle to cope with stress due to COVID-19 properly as well. At this point, we also need to consider the phenomenon of reversed causality. People with high depressive symptom levels seem to feel more helpless when coping with stress during the pandemic and perceive helplessness when coping with stress also predisposes to developing more depressive symptoms as they do not have suitable coping strategies. This can also result in a vicious circle of unsuitable coping strategies and depression or general mental distress. It is important to give this vulnerable group of oldest-old people, especially when already dealing with depressive symptoms, specific tools to improve their self-efficacy in coping with stress and thereby improve their mental health in the long run.

Our findings did not support our assumption that high depression and anxiety levels are associated with less perceived positive, self-efficient coping. We did not find significant associations of those factors. Furthermore, our model predicting negative coping explained twice as much variance in percent as did the model predicting positive coping with stress during the pandemic. It seems to be the case that the included psychosocial factors, more specifically depression, anxiety, somatization, social support, loneliness and resilience, are more crucial for the feeling of helplessness in coping with stress during the pandemic than for a positive, self-efficient way of coping. Anxiety, depression and loneliness are linked to a negative way of coping with stress but other factors seem to be important for the perception whether one feels self-efficient when coping. Those two concepts do not seem to be opposite sides of a coin but rather independent concepts that are influenced by different factors. Future studies should focus on potential influencing factors of positive, self-efficient coping with stress during critical times like the COVID-19 pandemic. The fact that resilience is associated with more perceived positive coping but not with less perceived negative coping also stresses the previous interpretation that we have two separate, distinct factors and not opposite poles of one dimension and that there must be more factors influencing it.

Loneliness is associated with both higher levels of positive and negative coping. Those two constructs are not necessarily mutually exclusive. The questionnaire assesses how often they felt either self-efficient or helpless in coping with stress during the last month. It seems that people who felt particularly lonely often felt helpless but at the same time experience situation where they felt self-efficient in stress coping. This could be due to the nature of problems and associated settings. While a person can feel very competent in coping with problems that they had in the past and/or have specific coping strategies for, there might be situations that are rather new and one has to adapt their coping strategy to a new setting, initially resulting in a feeling of helplessness. In the context of the COVID-19 pandemic, it could suggest that a strategy once including social contacts cannot be used due to the quarantine measures. More research is needed to clarify this finding and to investigate the psychological effects of the pandemic over its course and after a longer period of time.

### COVID-19-specific worries and personal threat

Contrary to some existing literature (Yu et al. 2020), our results indicate that social support may be associated with increased worries about COVID-19. At first glance, this seems contra-intuitive but when taking a closer look at the item wording you can see that the question regarding worries refers to more general worries about COVID-19, also concerning others. A possible explanation is that people who have more social support also have more social contacts resulting in having more persons to worry about (e.g. the grandchildren’s education, financial problems of children, higher risk for severe course of disease of friends and so on). That worries concern mostly others and not the older persons themselves is something that has been found in previous studies as well (Kuehner et al. 2020; Vannini et al. 2021). Thus, we suggest that social support can be a protective factor for distress in the very old population but in times of a pandemic having more loved ones might be associated with more worries about them and their future. Moreover, men may tend to worry more about COVID-19 compared to women. This is in contrary to some studies’ findings that women experience more stress due to COVID-19 (Hou et al. 2020) but may be explained partially by the higher mortality risk of men, and thus higher risk, when infected with the corona-virus (Barber & Kim 2021). Further studies are needed to clarify this finding.

Although the objective risk for a severe course of disease increases with higher age, our findings indicate that with increased age, individuals tend to experience less personal threat by COVID-19. Oldest-old individuals may feel that they have already lived their life and worry more about others than themselves which would be in line with studies suggesting that worries focus on others and less on the individual’s own health (Kuehner et al. 2020; Vannini et al. 2021). Higher levels of depression were associated with less perceived personal threat which is in line with finding that suggest that the nature of worries in depressive patients is more about the future, relationships and alike and less about personal physical harm (Diefenbach et al. 2001). Furthermore, there was a slightly higher chance of perceiving high personal threat when experiencing higher levels of somatization, which may hint to the fact that mental health factors influence the way one perceives the circumstance of the current pandemic.

The percentage of variance explained by the models is rather small, indicating that there are other factors substantially influencing the extent of distress due to COVID-19. Factors that might be worth exploring in future studies are personality characteristics like optimism or previous medical history.

### Limitations

While this study has several advantages like being one of the first studies that specifically investigates coping with stress in the high-risk group of the oldest-old population, there are some limitations, which need to be taken into account. Since we analyzed cross-sectional data, it is not possible to make statements about the causal direction of the association of included psychosocial variables and COVID-19-specific worries and perceived threat, positive and negative coping. Another factor worth mentioning is that most of the previous studies investigated specific coping strategies as opposed to the feeling of competence or incompetence in coping with stress as we do in our study. This makes comparing our findings directly with others challenging. Nevertheless, this understanding of the concept also brings advantages since we aimed to specifically investigate which factors influence the feeling of competence or incompetence when coping with stress regardless of specific strategies since the use and their benefits are highly individual (Bonanno & Burton 2013). One could argue that the extent of worries and perceived threat by COVID-19 plays a role in the way one feels when coping with stress. Sensitivity analysis showed that there is no significant contribution of those variables to explained variance in the before mentioned outcomes. Furthermore, we used a rather unusual sampling strategy but we see a strong advantage of it. The sample investigated in this study is unique and more challenging to win over to taking part in surveys or studies in general, especially during the COVID-19 pandemic. This sampling technique gave us the opportunity to investigate the sample of oldest-old people who have a high risk for a severe course of disease and are more likely to agree in taking part in the survey since they have taken part in our studies before compared to other oldest-old people. We also have a response rate of 52.12%. This is in line with the previous reasoning that this group is specifically difficult to win over for study participation. Nevertheless, there are no differences in age (*t* = 0.825, *p* = 0.410) nor gender (*x*^2^ = 0.702, *p* = 0.402) between responders (*n* = 197) and non-responders (*n* = 181). We also did not assess whether participants were chronically ill or in treatment but would argue that the vast majority of elderly people visits their general practitioner regularly due to some kind of illness (Linden et al. 1996). Still, this should be taken into account in future studies to be able to rule out the influence. Furthermore, we would like to note at this point that we chose to dichotomize the COVID-related worries and threat items in order to calculate a binary regression model. For an ordinal regression analysis due to originally ordinal item structure, the proportional odds assumption was not fulfilled and more complex models (e.g. generalized ordered logit models) with a requirement of larger sample sizes would have been required. Although we meet the most important sample size criteria for conducting regression analysis, we still have a rather small sample size and further research with bigger samples is needed.

## Conclusion

Our study allows us to shed light on the topic of coping with pandemic-related distress in the age group of the oldest-old people aged 79–100 years, which has often been left out in past investigations but represents a high-risk group of a severe course of disease when being infected with the Sars-CoV2 virus. Our findings show that in the group of the oldest-old people those with higher anxiety and depression levels are particularly vulnerable to feeling helpless in coping with stress and thus, those factors should be targeted in interventions aiming to improve coping skills in the oldest-old population. It seems to be especially important to also aim at boosting resilience to further trigger and strengthen the feeling of self-efficacy and competence in stress coping as it is crucial for improving and maintaining mental health in the very old individuals who feel burdened by the current COVID-19 pandemic. People who feel lonely should be supported in adapting their coping skill repertoire. Generally, mental health factors like depression may influence and shape the way older individuals experience distress due to the current pandemic. The current study’s findings elicit interesting starting points for future research aiming at maintaining and improving mental health in the very old population in times of public health crises and generally, in challenging and demanding situations that occur especially and more frequent with increased age.

## Data Availability

The data set used and analyzed during the current study is available from the corresponding author on reasonable request.

## References

[CR1] Aggarwal NT, Wilson RS, Beck TL, Rajan KB, de Leon M, Carlos F, Evans DA, Everson-Rose SA (2014). Perceived stress and change in cognitive function among adults 65 years and older. Psychosom Med.

[CR2] Arenaza-Urquijo EM, Przybelski SA, Machulda MM, Knopman DS, Lowe VJ, Mielke MM, Reddy AL, Geda YE, Jack CR, Petersen RC, Vemuri P (2020). Better stress coping associated with lower tau in amyloid-positive cognitively unimpaired older adults. Neurology.

[CR3] Barber SJ, Kim H (2021). COVID-19 worries and behavior changes in older and younger men and women. J Gerontol Ser B.

[CR4] Bastianon CD, Klein EM, Tibubos AN, Brähler E, Beutel ME, Petrowski K (2020). Perceived stress scale (PSS-10) psychometric properties in migrants and native Germans. BMC Psychiatry.

[CR5] Bonanno G, Burton C (2013). Regulatory flexibility: an individual differences perspective on coping and emotion regulation. Perspect Psychol Sci.

[CR6] Chmitorz A, Wenzel M, Stieglitz R-D, Kunzler A, Bagusat C, Helmreich I, Gerlicher A, Kampa M, Kubiak T, Kalisch R, Lieb K, Tüscher O (2018). Population-based validation of a German version of the brief resilience scale. PLoS ONE.

[CR7] Cohen S, Kamarck T, Mermelstein R (1983). A global measure of perceived stress. J Health Soc Behav.

[CR8] Cohen J, Cohen P, West S, Aiken L (2002). Applied multiple regression/correlation analysis for the behavioral sciences.

[CR9] Derogatis LR (1993) BSI brief symptom inventory. Administration, scoring, and procedures manual. https://ci.nii.ac.jp/naid/20000821855/en/

[CR10] Diefenbach GJ, McCarthy-Larzelere ME, Williamson DA, Mathews A, Manguno-Mire GM, Bentz BG (2001). Anxiety, depression, and the content of worries. Depress Anxiety.

[CR11] Franke G, Jaeger S, Glaesmer H, Barkmann C, Petrowski K, Brähler E (2017). Psychometric analysis of the brief symptom inventory 18 (BSI-18) in a representative German sample. BMC Med Res Methodol.

[CR12] Hardy SE, Concato J, Gill TM (2004). Resilience of community-dwelling older persons. J Am Geriatr Soc.

[CR13] Hou F, Bi F, Jiao R, Luo D, Song K (2020). Gender differences of depression and anxiety among social media users during the COVID-19 outbreak in China: a cross-sectional study. BMC Public Health.

[CR14] Hughes ME, Waite LJ, Hawkley LC, Cacioppo JT (2004). A short scale for measuring loneliness in large Surveys: results from two population-based studies. Res Aging.

[CR15] Kar N, Kar B, Kar S (2021). Stress and coping during COVID-19 pandemic: result of an online survey. Psychiatry Res.

[CR16] Kendel F, Spaderna H, Sieverding M, Dunkel A, Lehmkuhl E, Hetzer R, Regitz-Zagrosek V (2011). Eine deutsche Adaptation des ENRICHD Social Support Inventory (ESSI). Diagnostica.

[CR17] Khairulbahri M (2021). Understanding the first and the second waves of the COVID-19 in Germany: Is our social behavior enough to protect us from the pandemic?. J Sci Technol (WJST).

[CR18] Kuehner C, Schultz K, Gass P, Meyer-Lindenberg A, Dreßing H (2020). Psychisches befinden in der bevölkerung während der COVID-19-pandemie [mental health status in the community during the COVID-19-pandemic]. Psychiatr Prax.

[CR19] Kunzler AM, Chmitorz A, Bagusat C, Kaluza AJ, Hoffmann I, Schäfer M, Quiring O, Rigotti T, Kalisch R, Tüscher O, Franke AG, van Dick R, Lieb K (2018). Construct validity and population-based norms of the German brief resilience scale (BRS). Eur J Health Psychol.

[CR20] Leung DY, Lam T-H, Chan SS (2010). Three versions of perceived stress scale: validation in a sample of Chinese cardiac patients who smoke. BMC Public Health.

[CR21] Li R, Cooper C, Bradley J, Shulman A, Livingston G (2012). Coping strategies and psychological morbidity in family carers of people with dementia: a systematic review and meta-analysis. J Affect Disord.

[CR22] Linden M, Gilberg R, Horgas AL et al. (1996) Die inanspruchnahme medizinischer und pflegerischer hilfe im hohen alter. Die Berliner Altersstudie, 475–495 [article in German]

[CR23] Lopez J, Pérez-Rojo G, Noriega C, Carretero I, Velasco C, Martínez-Huertas J, Lopez-Frutos P, Galarraga Cristóbal L (2020). Psychological well-being among older adults during the Covid-19 outbreak: a comparative study of the young-old and the old-old adults. Int Psychogeriatr.

[CR24] Oles M, Oles P, Mookherjee S (2014). Coping style and quality of life in elderly patients with vision disturbances. J Ophthalmol.

[CR25] Orgeta V, Orrell M (2014). Coping styles for anxiety and depressive symptoms in community-dwelling older adults. Clin Gerontol.

[CR26] Raut N, Singh S, Subramanyam A, Pinto C, Kamath R, Shanker S (2014). Study of loneliness, depression and coping mechanisms in elderly. J Geriatr Ment Health.

[CR28] Robert Koch Institute (2020) Täglicher Lagebericht des RKI zur Coronavirus Krankheit-2019 (COVID-19) 31.05.2020 – Aktualisierter Stand für Deutschland. Situationsberichte. Accessed 18 May 2021. https://www.rki.de/DE/Content/InfAZ/N/Neuartiges_Coronavirus/

[CR29] Röhr S, Reininghaus U, Riedel-Heller SG (2020). Mental wellbeing in the German old age population largely unaltered during COVID-19 lockdown: results of a representative survey. BMC Geriatr.

[CR30] Russell D, Peplau LA, Cutrona CE (1980). The revised UCLA loneliness scale: concurrent and discriminant validity evidence. J Pers Soc Psychol.

[CR31] Schnell T, Krampe H (2020). Meaning in Life and self-control buffer stress in times of COVID-19: moderating and mediating effects with regard to mental distress. Front Psych.

[CR32] Smith MM, Saklofske DH, Keefer KV, Tremblay PF (2016). Coping strategies and psychological outcomes: the moderating effects of personal resiliency. J Psychol.

[CR33] Tagay Ö, Karataş Z, Bayar O, Savi F (2016). Resilience and life satisfaction as the predictors of general self-efficacy. Glob J Guid Couns Sch Curr Perspect.

[CR34] Taylor S, Landry CA, Paluszek MM, Fergus TA, McKay D, Asmundson GJG (2020). COVID stress syndrome: Concept, structure, and correlates. Depress Anxiety.

[CR35] Tomás JM, Sancho P, Melendez JC, Mayordomo T (2012). Resilience and coping as predictors of general well-being in the elderly: a structural equation modeling approach. Aging Ment Health.

[CR36] Vannini P, Gagliardi GP, Kuppe M, Dossett ML, Donovan NJ, Gatchel JR, Quiroz YT, Premnath PY, Amariglio R, Sperling RA, Marshall GA (2021). Stress, resilience, and coping strategies in a sample of community-dwelling older adults during COVID-19. J Psychiatr Res.

[CR37] Verity R, Okell LC, Dorigatti I, Winskill P, Whittaker C, Imai N, Cuomo-Dannenburg G, Thompson H, Walker PGT, Fu H, Dighe A, Griffin JT, Baguelin M, Bhatia S, Boonyasiri A, Cori A, Cucunubá Z, FitzJohn R, Gaythorpe K, Ferguson NM (2020). Estimates of the severity of coronavirus disease 2019: a model-based analysis. Lancet Infect Dis.

[CR38] Warttig SL, Forshaw MJ, South J, White AK (2013). New, normative, English-sample data for the short form perceived Stress Scale (PSS-4). J Health Psychol.

[CR39] Welzel FD, Schladitz K, Förster F, Löbner M, Riedel-Heller SG (2021). Gesundheitliche Folgen sozialer Isolation: Qualitative Studie zu psychosozialen Belastungen und Ressourcen älterer Menschen im Zusammenhang mit der COVID-19-Pandemie. Bundesgesundheitsblatt - Gesundheitsforschung - Gesundheitsschutz.

[CR40] Wilson R, Arnold S, Schneider J, Li Y, Bennett D (2007). Chronic distress, age-related neuropathology, and late-life dementia. Psychosom Med.

[CR41] Yu H, Li M, Li Z, Xiang W, Yuan Y, Liu Y, Li Z, Xiong Z (2020). Coping style, social support and psychological distress in the general Chinese population in the early stages of the COVID-19 epidemic. BMC Psychiatry.

